# Evaluation of brachycephalic obstructive airway syndrome breeding test results in Finland from 2017 to 2022

**DOI:** 10.1186/s13028-024-00755-9

**Published:** 2024-07-18

**Authors:** Liisa Iiris Onerva Lilja-Maula, Katariina Helena Mäki, Mimma Kristiina Aromaa, Minna Marjaana Rajamäki

**Affiliations:** 1 Department of Equine and Small Animal Medicine, Faculty of Veterinary Medicine, University of Helsinki, P. O. Box 57, 00014 Helsinki, Finland; 2 Finnish Kennel Club, Valimotie 17-19, Helsinki, Finland; 3 International Partnership For Dogs, Helsinki, Finland

**Keywords:** Animal welfare, Exercise test, Flat-faced dogs, Inheritance, Upper respiratory disease

## Abstract

**Background:**

Brachycephalic obstructive airway syndrome (BOAS), observed in many flat-faced dog breeds, is one of the most urgent welfare problems in pedigree dogs. Various breeding schemes against BOAS have been implemented in many countries during recent years, but their impact on breed health remains unknown. The BOAS breeding test, used by the Finnish Kennel Club (FKC), includes an exercise component with a recovery assessment, BOAS grading by a veterinarian that evaluates upper respiratory signs before and after exercise, and a nostril stenosis assessment. The aim of our study was to evaluate BOAS breeding test results and estimate the heritability of the BOAS grade using parent–offspring regression from FKC data collected during 2017–2022.

**Results:**

The majority (80%) of dogs (n = 957) participating in FKC BOAS testing were English Bulldogs, French Bulldogs, and Pugs. In 2022, 89–100% of the litters from these three breeds registered with the FKC had at least one parent tested for BOAS. The proportion of dogs failing the exercise test was highest in English Bulldogs (11%), followed by French Bulldogs (4%) and Pugs (3%). In these three breeds, moderate to severe BOAS signs were reported in 28%, 22% and 30% of dogs, respectively. The proportion of moderate to severe nostril stenosis was highest (71%) in Pugs, followed by French Bulldogs (55%), and English Bulldogs (40%). Estimates of heritability for BOAS grade were separately calculated for these three breeds and for all dogs, and the estimates were moderate to high, ranging from 0.39 to 0.58.

**Conclusions:**

The exercise test alone did not sufficiently identify dogs with moderate to severe BOAS signs. To better consider the complex nature of BOAS and breed differences, exercise tolerance, the severity of upper respiratory signs (BOAS grade) and nostril stenosis should all be assessed together in breeding animals. The heritability estimates for veterinary-assessed BOAS grade indicated that BOAS grade could be used in selective breeding to obtain less-affected offspring.

## Background

Conformation-related health issues in pedigree dogs have gained vast publicity worldwide during recent years. Brachycephalic obstructive airway syndrome (BOAS) in particular, directly linked to short facial anatomy seen in many popular breeds, such as the French Bulldog, Pug, and English Bulldog, causes major welfare problems [1–3]. Dogs with the extreme brachycephalic phenotype suffer from respiratory distress, exercise and heat intolerance, sleep disturbances, and gastrointestinal problems that can vary from mild to life-threatening [4–8]. Although the severe nature of BOAS-related signs have been recognized for decades, and the need for breeding towards less extreme facial features has been evident, BOAS prevalence remains high and reports on outcomes of breeding schemes against BOAS are lacking [7, 9, 10].

Approximately 800 000 dogs currently live in Finland, around 73% of which are purebreds registered with the Finnish Kennel Club (FKC) [11]. This large proportion of registered dogs offers an excellent possibility to influence breed health and evaluate the outcomes of various health screening programmes. FKC member breed clubs can implement voluntary or mandatory breed-specific health tests for breeding approval and thereafter for litter registration with the FKC. All health test results are publicly available from the FKC website. In 2017, the FKC launched the BOAS breeding test for brachycephalic dogs based on an English Bulldog health study conducted at the University of Helsinki, Finland during 2014–2015 [12]. The test protocol was later also studied in Pugs and French Bulldogs [13]. The FKC test includes an exercise component with recovery and body temperature measurements, veterinary-assessed BOAS severity grading, and an evaluation of nostril stenosis. In addition, the craniofacial ratio (CFR) is measured as an optional feature to evaluate muzzle length. [14] The exercise component includes a brisk 1000 m walk on a leash with a time limit defining the threshold that a dog must be able to perform. Additionally, the test includes an evaluation of recovery and a measurement of rectal temperature [12, 13]. Clinical BOAS grading is performed by evaluating audible upper respiratory noises and any signs of respiratory distress before and after exercise using a four-grade scale from none to severe [12]. The BOAS grading used in our present study is modified from the respiratory function grading (RFG) scheme first presented by Liu et al. [15]. Results of the exercise component (pass or fail) and BOAS grading (no, mild, moderate, or severe signs) are stored in the FKC public breeding database. At the time of the study, the English Bulldog, French Bulldog, Pug, and Continental Bulldog breed clubs in Finland had mandatory BOAS screening for one or both parents of the litter prior to breeding. Currently, also Pekingese, Griffons, and Shih Tzu breed clubs have mandatory testing. The exercise component must be passed in all these breeds, and the Pekingese and Shih Tzu clubs forbid using dogs with moderate or severe BOAS signs. Other brachycephalic breed clubs in Finland do not have mandatory BOAS testing before breeding, but the FKC test is also available to them.

Raising awareness about the harmful consequences of BOAS, legislative actions, and implementation of various breeding selection tools have been conducted in many countries [9]. Recently, the world canine organization, Fédération Cynologique Internationale (FCI) has encouraged its member kennel clubs to implement the RFG scheme [15, 16] for brachycephalic dogs [17]. The UK Kennel Club has used the RFG scheme from 2019 onwards and advises that dogs with severe BOAS signs should not be used for breeding [18]. Among countries currently using the RFG scheme, the Swedish and Norwegian Kennel Clubs have forbidden the breeding of brachycephalic dogs with moderate and severe BOAS signs [19, 20]. In Germany, passing an exercise test has been mandatory for Pugs used in breeding since 2009 [21]. In 2020, the Finnish Food Authority published a report on possible actions to regulate the breeding of brachycephalic dogs to efficiently improve breed health [22]. According to this proposal, clinically assessed BOAS and nostril stenosis grades, exercise tolerance, and muzzle length should be used as breeding criteria in the future, to reduce BOAS prevalence.

Despite all the actions taken against BOAS, it remains unclear whether selective breeding using a less affected phenotype will be enough or whether crossbreeding is the only viable option to obtain healthier brachycephalic dogs. No studies reporting heritability estimates and possible benefits of BOAS screening programmes have been published. Most studies describing BOAS signs and their prevalences have additionally been conducted in study populations recruited for research, not in actual breeding populations. Therefore, the main objectives of our present study were to evaluate the results gained from the FKC BOAS breeding test from years 2017–2022 and to estimate the heritability for clinically graded BOAS signs.

## Methods

### Study population

All BOAS test data from the FKC register from 2017–2022 were used for BOAS test result analysis. For BOAS grade heritability calculations, data from 2017 until April 2023 were available for analysis. Only the exercise test results were available from all the dogs that had participated in FKC BOAS screening test, as originally it was the only mandatory result to be electronically recorded. Other results (BOAS grade, nostril stenosis grade, body temperature) have all been performed according to FKC protocol, but available for analyses only from those dogs, whose paper forms have been sent to FKC. A list of brachycephalic breeds that were eligible to attend the FKC BOAS test, their place on the list of the 100 most popular dog breeds in Finland, and the number of registrations in 2017–2022 are presented in Table 1.

**Table 1:** Brachycephalic breeds eligible for FKC BOAS breeding test and popularity of these breeds

Breed	Year, registration n (place on list of 100 most popular dog breeds)					
	2017	2018	2019	2020	2021	2022
French Bulldog	472 (25.)	415 (28.)	415 (29.)	358 (37.)	337 (45.)	300 (48.)
Pug	346 (36.)	336 (37.)	263 (48.)	209 (68.)	198 (77.)	135 (92.)
Boston Terrier	264 (53.)	205 (63.)	199 (66.)	167 (81.)	168 (85.)	152 (83.)
English Bulldog	198 (68.)	121 (98.)	149 (84.)	137 (94.)	88	87
Griffons	122	159	143	149	153	117
Shih Tzu	112	100	51	79	66	115
Japanese Chin	60	65	42	76	69	84
King Charles Spaniel	23	24	29	29	34	22
Affenpinscher	35	12	27	27	19	4
Pekingese	24	24	29	29	34	22
Continental Bulldog*						4
Altogether	1656^a^	1461	1347	1260	1166	1037^b^
All FKC breeds	46 495	45,716	45,096	48,932	57,745	48 627

FKC: Finnish Kennel Club; BOAS: brachycephalic obstructive airway syndrome

^*^Breed recognised by Fédération Cynologique Internationale in 2022

^a^3.6% of all registrations

^b^2.1% of all registrations

### The Finnish Kennel Club brachycephalic obstructive airway syndrome test protocol

The test consists of an evaluation of a dog’s exercise tolerance including recovery, BOAS grade, and nostril stenosis grade, and CFR as an optional measurement. The test can be repeated. The FKC BOAS test is supervised by named veterinarians, who are trained by one of the authors (LLM or MA). A total of 11 Finnish veterinarians have permission to supervise the FKC BOAS testing. During the test, the supervising veterinarian records information on the dog and location of the test place on a paper form. After testing, the exercise test result and the BOAS grade are stored in the FKC electronic database and a copy of the paper form is requested to be sent to the FKC.

Initially (2017–2020), only the exercise test result, i.e. passed, failed, or discontinued, was recorded in the FKC public breeding database. Later, from 2021 onwards, the BOAS grade has also been stored in the public breeding database. Nostril stenosis and CFR data are currently available only in paper form.

The exercise component of the test is based on our previous studies [12, 13], and the protocol is available on the FKC website [14]. To pass the test, a dog must briskly walk or trot on the leash, guided by its owner or person selected by the owner, for 1000 m within a required breed-specific time limit and recover sufficiently from it to pre-walk status (heart rate, body temperature, general condition) within 15 min. The walking time limit for passing the test is 11 min for Pugs, French Bulldogs, and Boston Terriers and 12 min for other breeds. Rectal temperature is measured before and after the exercise and after the recovery period. The dog cannot participate in the test if its pre-exercise temperature is above 39.3 °C, and the test is failed if its post-exercise temperature rises above 39.5 °C. Pre- and post-exercise temperature limits are based on our previous studies on brachycephalic and control dogs [12, 13]. The test is preferably held indoors, and if outdoors, the weather must be dry. Temperature at the test site must be 15–25 °C. If the dog experiences respiratory difficulties during the test, it is discontinued and considered failed due to BOAS-related reasons. The test can also be discontinued for other than BOAS-related issues, such as orthopaedic or motivation problems, in which case the test is not regarded as failed.

The four-grade scale for clinical BOAS signs (no, mild, moderate, or severe signs) used in the FKC BOAS test is based on audible upper respiratory sounds and signs of respiratory effort and distress before and after exercise, as described in our first study [12], and is available in its current form on the FKC website [23].

Nostril stenosis is evaluated visually as earlier described [7, 15] using four-grade scale (open, mild, moderate, or severe stenosis) and is available on the FKC website [24].

CFR is measured as an optional feature and is calculated by dividing muzzle length by cranial length, as previously described [25]. The protocol is available from the FKC website [26].

### Data analysis

Descriptive statistics are used for the exercise results, BOAS grade, nostril stenosis grade, and temperature and CFR results.

As only the parent IDs of the tested dogs were available, and not the entire pedigrees of the breeds, the parent–offspring regression was used to calculate a rough estimate of heritability for the BOAS grade. The Wsys-L programme was used in the estimation. The data consisted of the ID numbers of the dogs and their parents, along with the breed, sex, birth date, test date, and age at testing. The parent–offspring regression model is y = a + bX + e, where y = the BOAS grade of the offspring, b = regression coefficient, and X = the BOAS grade of a parent or the mean of both parents. Heritability corresponds to the regression coefficient when X is the mean of the parents’ BOAS grade. When X is the BOAS grade of only one parent, heritability is obtained by multiplying the regression coefficient by two.

Heritability was first estimated using data from the three breeds for which the BOAS test has previously been studied: the English Bulldog, French Bulldog, and Pug. From these data, heritability was estimated for two separate datasets. The first dataset included all parent–offspring pairs in which both the offspring and one of the parents had been tested (dataset 1). The second dataset consisted only of pairs in which both parents had a BOAS grade (dataset 2). In this dataset, the mean of the parents was used as the parent score. Finally, heritability was estimated for a dataset including all breeds (dataset 3). This dataset included parent–offspring pairs with one tested parent. It was not necessary to analyse dataset 2 for all breeds, as all pairs with both parents tested belonged to the three abovementioned breeds.

P-values < 0.05 were considered statistically significant.

### Ethical statement

BOAS breeding test results were obtained from the FKC register. All owners whose dogs participate in FKC BOAS testing sign an informed consent for their dogs’ results to be published in the public FKC breeding database and further used for research. The retrospective data obtained from the FKC have been handled in accordance with regulations for data processing at the FKC and the University of Helsinki, Finland.

## Results

The number of French Bulldog, Pug, and English Bulldog litters in which at least one parent or both parents have been BOAS tested in 2018, one year after test implementation, and in 2022 are presented in Table 2.

**Table 2:** FKC registered litters of French Bulldogs, Pugs, and English Bulldogs with BOAS screened parents

Breed	Year 2018^a^		Year 2022^b^	
	At least one parent screened, n (%)	Both parents screened, n (%)	At least one parent screened, n (%)	Both parents screened, n (%)
French Bulldog	15/86 (17)	2/86 (2)	57/64 (89)	20/64 (31)
Pug	29/85 (34)	2/85 (2)	36/38 (95)	13/34 (34)
English Bulldog	8/30 (27)	2/30 (7)	15/15 (100)	14/15 (93)

FKC: Finnish Kennel Club; BOAS: brachycephalic obstructive airway syndrome

^a^One year after the brachycephalic obstructive airway syndrome (BOAS) test was implemented, and none of the breeds had mandatory screening

^b^BOAS screening mandatory for at least one parent in French Bulldogs (since 2022) and Pugs (since 2021, for both parents since 2024), and for both parents in English Bulldogs (since 2019)

### Exercise test

Exercise test results from 957 dogs are presented by breed in Table 3. Overall, 94% of tested dogs passed the exercise component, 5% failed it, and 1% discontinued the test for other than BOAS-related issues. As Pekingese dogs have extremely short legs, exercise test time limits were not initially used for this breed during 2017–2022, and therefore their exercise test results are not reported here. In addition, the results of two Affenpinschers are not presented to ensure anonymity.

**Table 3:** Exercise test results from FKC BOAS breeding test between 2017–2022

Breed (n)	Passed n (%)	Failed n (%)	Discontinued due to other than BOAS related issues n (%)
French Bulldog (311)	300 (96)	11 (4)	0
English Bulldog (245)	213 (87)	28 (11)	4 (2)
Pug (206)	197 (96)	6 (3)	3 (1)
Griffons (93)	91 (98)	0	2 (2)
Boston Terrier (50)	50 (100)	0	0
King Charles Spaniel (21)	19 (90)	0	2 (10)
Shih Tzu (19)	19 (100)	0	0
Japanese Chin (12)	12 (100)	0	0
Altogether (957^a^)	901 (94)	45 (5)	11 (1)

FKC: Finnish Kennel Club; BOAS: brachycephalic obstructive airway syndrome

^a^For dogs that participated twice (n = 27), only one result from the first test visit is included

Post-exercise temperature results were available for 569 dogs. A total of 37/569 (7%) dogs had elevated post-exercise temperatures (over 39.5 °C), and ten of these had temperatures over 40.0 °C. Approximately half of the dogs with an elevated temperature were dogs with no or only mild BOAS signs (19/37) and half were dogs with moderate or severe signs (18/37), while 17 were females and 20 were males. Elevated body temperature was most common in the Pekingese, seen in 4/7 (57%) tested dogs, and in the English Bulldog (23/195; 12%). In addition, 4/128 (3%) Pugs, 3/33 (9%) Griffons, 2/134 (1.5%) French Bulldogs, and 1/17 (6%) King Charles Spaniel had elevated temperatures.

### Brachycephalic obstructive airway syndrome grades

**Fig. 1:**
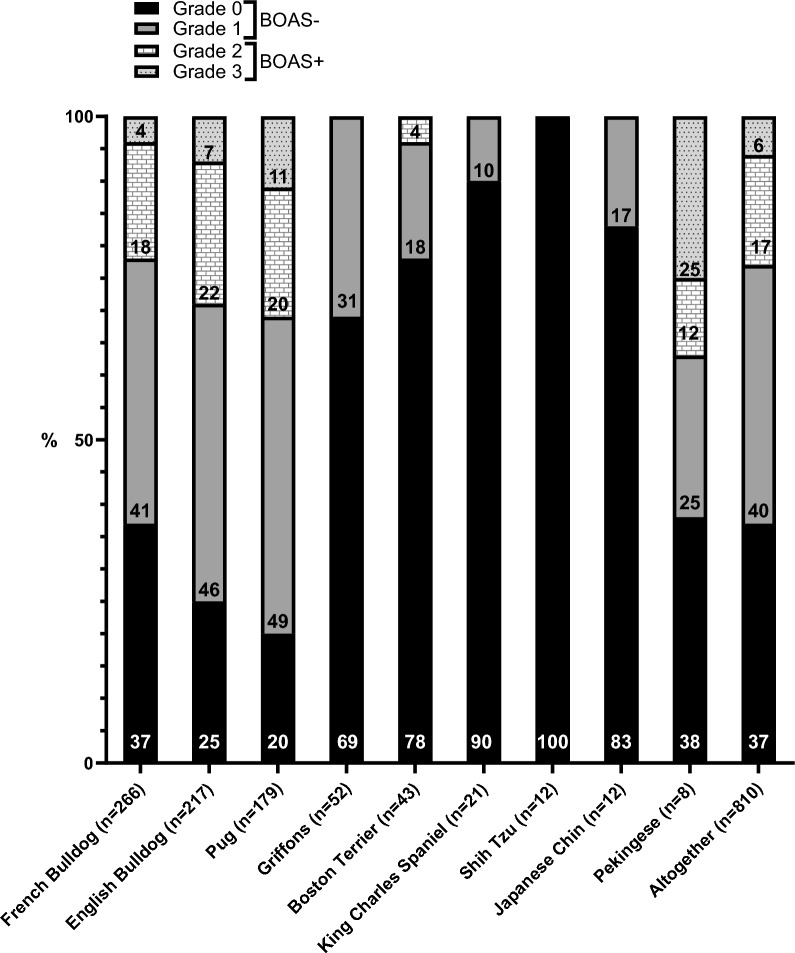
BOAS severity grades from FKC BOAS breeding test between 2017 and 2022. BOAS: brachycephalic obstructive airway syndrome; FKC: Finnish Kennel Club. Grade 0 = no, Grade 1 = mild, Grade 2 = moderate, Grade 3 = severe BOAS signs. For dogs that participated twice (n = 6), only one result from the first test visit is included

BOAS grades from 810 dogs are presented by breed in Fig. 1. The results of two Affenpinschers are not presented to ensure anonymity. Altogether, when categorizing grades 0 and 1 into the BOAS- class (i.e. dogs that do not show clinically significant BOAS signs) and grades 2 and 3 into the BOAS + class (i.e. clinically affected), as previously suggested [15], 77% of dogs that participated in the testing were classified as BOAS-.

### Nostril stenosis

**Fig. 2:**
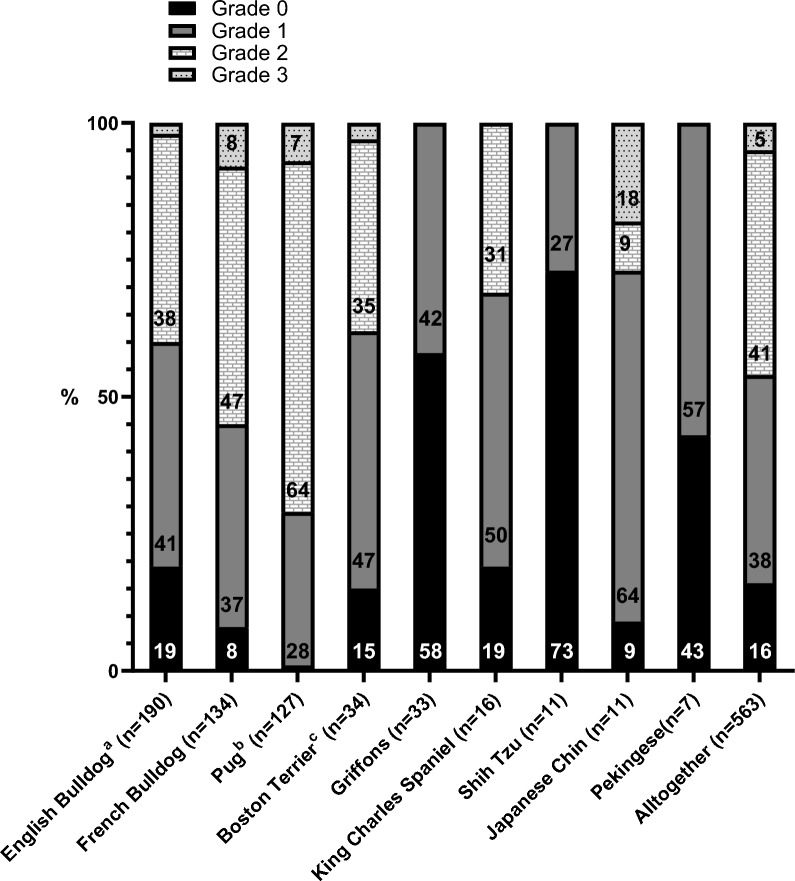
Nostril stenosis grades from FKC BOAS breeding test between 2017 and 2022. FKC: Finnish Kennel Club; BOAS: brachycephalic obstructive airway syndrome. Grade 0 = open, Grade 1 = mild, Grade 2 = moderate, Grade 3 = severe nostril stenosis. For dogs that participated twice (n = 5), only one result from the first test visit is included. ^a^Grade 3: 2%; ^b^Grade 0: 1%; ^c^Grade 3: 3%

Nostril stenosis grading results for 563 dogs are presented by breed in Fig. 2. Nearly half of the dogs (46%) had moderately or severely stenotic nares.

### Craniofacial ratio

**Fig. 3:**
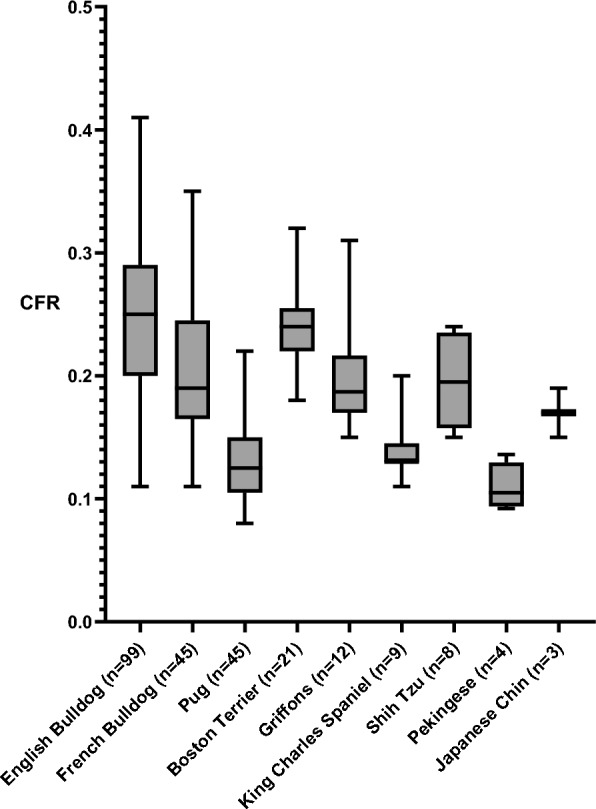
Craniofacial ratio (CFR) results from FKC BOAS breeding test between 2017 and 2022. FKC: Finnish Kennel Club; BOAS: brachycephalic obstructive airway syndrome. For dogs that participated twice (n = 4), only one result from the first test visit is included

CFR results for 246 dogs are presented by breed in Fig. 3. If using the Finnish Food Authority 2020 recommendation for muzzle length (CFR at least 0.33) [22], only ten English Bulldogs and three French Bulldogs out of 246 dogs would pass the limit.

### Heritability estimates for brachycephalic obstructive airway syndrome grade

The characteristics of 845 dogs used for BOAS grade heritability calculations are presented in Table 4. If the dog participated twice (n = 7), only the most recent result was included. Estimates of heritability for the different datasets were moderate to high, ranging from 0.39 to 0.58 (Table 5). The regression coefficients suggest that the offspring BOAS grade increases by 0.24 to 0.39 points for every one-point increase in the parent BOAS grade.

**Table 4:** Characteristics of the dogs included in the calculation of BOAS grade heritability

Breed (n)	Gender		Age at time of testing (months; mean, range)
	Female n (%)	Male n (%)	
French Bulldog (277)	159 (57%)	118 (43%)	35.4 (18–119)
English Bulldog (221)	134 (61%)	87 (39%)	31.8 (16–92)
Pug (182)	91 (50%)	91 (50%)	35.2 (18–112)
Griffons (56)	33 (59%)	23 (41%)	39.3 (18–132)
Boston Terrier (55)	36 (65%)	19 (35%)	36.2 (18–132)
King Charles Spaniel (21)	13 (62%)	8 (38%)	48.5 (23–112)
Shih Tzu (13)	7 (54%)	6 (46%)	43.2 (19–114)
Japanese Chin (12)	8 (67%)	4 (33%)	36.8 (21–75)

BOAS: brachycephalic obstructive airway syndrome

**Table 5:** Heritability estimates for BOAS severity grades from FKC

Dataset	Number of parent–offspring pairs	Heritability (sd)	p-value (t test)
1^a^	304	0.48 (0.07)	0.000
2^b^	68	0.39 (0.16)	0.019
3^c^	338	0.58 (0.06)	0.000

BOAS: brachycephalic obstructive airway syndrome; FKC: Finnish Kennel Club

^a^Including parent–offspring pairs of English Bulldogs, French Bulldogs, and Pugs with at least one BOAS-graded parent

^b^Including parent–offspring pairs of English Bulldogs, French Bulldogs, and Pugs with both parents BOAS graded

^c^Including parent–offspring pairs of all brachycephalic dogs (English Bulldogs, French Bulldogs, Pugs, Griffons, Boston Terriers, King Charles Spaniels, Shih Tzus, Japanese Chins, and Pekingese) with at least one BOAS-graded parent

## Discussion

In the present study, we evaluated the BOAS breeding test results obtained from the FKC database during 2017–2022. Our results showed that the exercise test alone was not sufficient to effectively exclude BOAS-affected dogs from breeding. The heritability estimates for BOAS grade, which describes the severity of upper respiratory noises and respiratory effort before and after exercise, indicated that BOAS grade could be used in selective breeding against BOAS to gain genetic improvement.

The 1000 m exercise test was failed by 11% of English Bulldogs. Only 4% and 3% of French Bulldogs and Pugs failed the test, respectively, and none of the other breeds failed. We have previously studied the 1000 m exercise test for BOAS severity assessment in English Bulldogs, French Bulldogs, and Pugs [12, 13, 27]. As the number of BOAS + (i.e. dogs with moderate or severe respiratory signs) English Bulldogs, French Bulldogs, and Pugs in the present study was clearly larger, ranging from 22 to 30%, than those failing the exercise component, it is evident that the test did not identify affected dogs as efficiently as desired. However, we note that, altogether, only 6% of tested dogs showed severe BOAS signs. It is likely that more severely affected dogs seldom participate in breeding tests, especially when not mandatory. In our previous studies, most, but not all, BOAS + dogs failed the exercise test [12, 13], which is in line with findings of a submaximal treadmill test for Pugs [10]. This discrepancy in the number of BOAS + dogs failing the test in our studies may be due to the different test environments. In our previous studies, dogs were walked with a researcher in a clinic environment, whereas dogs in the FKC test are walked with their owners in more casual surroundings. As signs of BOAS can easily be exacerbated by stress and other environmental factors, dogs, especially those showing moderate signs, may have performed better in the FKC test with their owner present and a more relaxed environment. As we previously recommended, tightening the exercise test limits, especially the recovery time limit [13], could increase the proportion of excluded BOAS + dogs. Nevertheless, as exercise and heat intolerance along with delayed recovery are key features seen in BOAS-affected dogs [6], and some BOAS- (i.e. dogs showing no or only mild respiratory signs) dogs can also suffer from these features, it is advisable to evaluate them together with other aspects of BOAS in breeding animals.

In addition to exercise tests, other non-invasive methods for evaluating BOAS severity, such as the grading of respiratory signs and external conformational characteristics, have previously been reported [15, 16, 25, 28] and could be used as breeding selection tools. In our present study, 28% of English Bulldogs, 22% of French Bulldogs, and 30% of Pugs were BOAS + , i.e. showed moderate or severe respiratory signs, which is in line with our previous results [12, 13], while no BOAS + dogs of some other breeds were recorded. The proportion of severely affected dogs was highest in the Pekingese (25%) and Pugs (11%), but the number of tested Pekingese dogs was very small. When comparing our present results with available data from the UK Kennel Club RFG scheme [29], from which our grading protocol is modified, the percentage of BOAS + dogs in our stock of English Bulldogs, French Bulldogs, and Pugs is slightly higher. In UK data, 18% of English Bulldogs, 16% of French Bulldogs, and 22% of Pugs were classified as BOAS + . Although differences in populations are possible, the longer exercise duration in our tests, around 11 min compared to 3 min used in the RFG scheme, can also reflect the larger BOAS + proportion. The effect of the longer exercise duration was shown in a treadmill test for Pugs, where every 5 min of exercise increased the proportion of dogs classified as BOAS + using a modified RFG scheme [10]. Also, the mandatory testing requirement before breeding in the FKC may influence the BOAS results seen in these three breeds in our study. It should also be kept in mind that an evaluation of respiratory noises and respiratory effort in these grading schemes is always subjective, and grading always varies more as the number of evaluators increases. Therefore, the number of evaluators in Finland has been kept small. In the future, machine learning for evaluating respiratory sounds could offer new, objective ways of assessing BOAS severity [30]. As these clinical respiratory sign grading schemes for BOAS are currently enforced in many countries and recommended by the FCI, it is vital to understand whether genetic improvement can be achieved by using them as a breeding selection tool. In present study, we estimated BOAS grade heritability by using parent–offspring regression, which is a traditional method to determine the heritability of phenotypic traits, and is commonly used when extended pedigree information is not available. Heritability itself is an indication of the proportion of genetic variation in a certain trait. Based on heritability, we can see whether the measure of the trait is capable of detecting genetic differences between animals and, therefore, whether breeding for the trait is possible using this measure. The magnitude of the heritability estimates in our study were moderate to high, indicating that veterinary-assessed BOAS grades can be used in selective breeding and that it is possible to achieve genetic improvement based on this selection. This finding is very encouraging, as the concern for brachycephalic dog welfare remains high.

Of the conformational factors, moderate or severe nostril stenosis was seen in nearly half (46%) of the evaluated dogs, but this percentage varied considerably between breeds. The highest proportion of moderately or severely stenotic nares (71%) was seen in Pugs, while the proportion varied between 0 to 55% in all the other breeds. CFR measurements were very low, as expected, and only 13 dogs, mainly English Bulldogs, would pass the Finnish Food Authority’s recommended limit of 0.33. Although nostril stenosis and CFR have been shown to correlate with BOAS severity, clear discrepancies exist between studies, and their sole use for BOAS breeding testing is therefore unadvisable [10, 25, 28, 31]. In the Netherlands, brachycephalic dog breeding has practically ceased due to a legislative CFR requirement of 0.3 [32]. Despite differences in studies regarding the usefulness of nostril stenosis grading and CFR for screening BOAS affectedness among brachycephalic breeds, breeding towards longer noses and more open nostrils is reasonable without question [10].

When evaluating the different aspects of BOAS screening test results in our present study, 11% of English Bulldogs failed the exercise component, but only 2% would have been excluded from breeding due to severe nostril stenosis and 7% due to severe BOAS signs. Severe nostril stenosis was seen in 7% of Pugs, and in 8% of French Bulldogs, while only 3% to 4% failed the exercise test. Thermoregulation problems during exercise were seen especially in English Bulldogs and Pekingese and occurred also in dogs with no or mild BOAS signs (i.e. BOAS- dogs). These findings further emphasize the need to evaluate and combine several aspects of BOAS when making breeding selection recommendations and to recognize breed differences among brachycephalic breeds. Based on our results, dogs with moderate and severe BOAS signs and those failing the exercise test could be excluded from breeding without limiting the genetic pool too much. However, excluding dogs with moderate to severe nostril stenosis would not be possible in several breeds without including dogs of other breeds in the breeding program.

The huge worldwide popularity of brachycephalic dogs has highlighted the welfare problems seen in these breeds. However, our results show a clear decline in the popularity of brachycephalic breeds in Finland between 2017 and 2022, as registrations of brachycephalic dogs have decreased from 3.6% to 2.1% of all registrations made and their placements on lists indexing the top 100 popular dog breeds have dropped dramatically. This phenomenon in Finland may be due to public discussion and better recognition of health issues seen in these breeds. The FKC, along with the Finnish French Bulldog club, has also initiated in 2023 a crossbreeding programme for French Bulldogs [33]. Brachycephalic breed clubs have also actively participated in our previous BOAS studies [12, 13, 27] and implemented mandatory testing. Our data show that mandatory testing is needed to effectively increase the proportion of BOAS-screened dogs. In 2018, one year after test implementation, none of the English Bulldog, French Bulldog, or Pug breed clubs had mandatory BOAS testing for breeding approval, and the number of litters with at least one BOAS-screened parent varied between 17 and 34%. In comparison, this proportion was 89–100% in 2022 due to mandatory testing, albeit excluding foreign dogs. Raising awareness among breeders and puppy buyers along with implementing breeding selection tools has clearly impacted the popularity of and health awareness concerning brachycephalic breeds in Finland.

Limitations of our study include the relatively small sample size and the lack of breed pedigree data, which prevented us from using more robust methods, such as the Best Linear Unbiased Prediction (BLUP) animal model, for more precise heritability estimates. In addition, the use exercise test and BOAS grading has not been studied in other than the three most common brachycephalic breeds, i.e. the English Bulldog, French Bulldog, and Pug, and might therefore not reflect the BOAS severity in other brachycephalic breeds in the same manner. Therefore, BOAS test results gained from other than the three previously studied breeds should be interpreted with caution. It should also be kept in mind, that the numbers of tested individuals in these other breeds were small, and as the test was not mandatory for these breeds at the time of the study period, it is likely that more severely affected animals were not brought to the evaluation, therefore providing overly optimistic results. For these other breeds, more breed-specific information is needed before deciding on how well the exercise testing and BOAS grading works for their breeding evaluation. However, the vast majority of dogs in our present study were English Bulldogs, French Bulldogs, and Pugs, and hereditary calculations were performed separately for all the dogs and for these three previously studied breeds.

Although our results indicate that clinical BOAS grading is useful to gain healthier offspring by selective breeding, it should be kept in mind that certain brachycephalic breeds also have other urgent health issues, such as skin, ocular, and skeletal problems [34–37]. Vertebral anomalies and intervertebral disc disease in particular, seen commonly in French Bulldogs, need to be addressed [38, 39]. Testing only for BOAS should therefore not to be used as the definition for a healthy brachycephalic dog. In Finland, radiographic screening for breeding purposes, of hip and elbow dysplasia, elbow incongruity, vertebral anomalies, and intervertebral disc calcifications is available depending on the breed [40].

## Conclusions

Our results indicated that genetic improvement can be obtained and the severity of BOAS signs in offspring reduced by using clinical BOAS grading, which evaluates upper respiratory signs before and after exercise. The exercise test alone was not efficient enough to identify all dogs with moderate or severe BOAS signs. However, as poor exercise and heat tolerance are major signs seen in BOAS-affected dogs and can also be seen in dogs classified as having no or mild signs, exercise testing should be performed together with clinical BOAS grading. In addition, as moderate to severe nostril stenosis was highly prevalent, and stenotic nares are one of the primary anatomical abnormalities seen in BOAS, also the nostril stenosis grade should be assessed in breeding animals. Mandatory testing before breeding approval is also needed to effectively increase the portion of BOAS-tested dogs. In the future, when more BOAS screening data from various countries and breeds becomes available, heritability and outcome of the breeding programmes should be further evaluated to verify the genetic improvement gained by selecting against BOAS.

## Data Availability

The data that support the findings of this study are available from the FKC, but restrictions apply to data availability, as the data were used under licence for the current study, and so are not publicly available. However, data are available from the authors upon reasonable request and with permission from the FKC.
